# Supplementary material for: Machine learning-based mortality prediction in critically ill patients with hypertension: comparative analysis, fairness, and interpretability
Source: Front Artif Intell. 2025 Dec 11;8:1686378. doi: 10.3389/frai.2025.1686378 (PMC12738824; doi:10.3389/frai.2025.1686378)
Supplement: Supplementary file 1 [file Presentation_1.pdf]

SUPPLEMENTARY MATERIAL

Supplementary Table 1. Summary of cross-validation results for model performance on the validation set in hypertension mortality prediction using all features.

**Models / Metrics**	**Accuracy, 95% CI**	**AUROC, 95% CI**	**Sensitivity, 95% CI**	**Specificity, 95% CI**	**F1 Score, 95% CI**
Logistic Regression	0.866 [0.858, 0.874]	0.943 [0.938, 0.949]	0.847 [0.839, 0.855]	0.881 [0.865, 0.897]	0.864 [0.854, 0.873]
Random Forest	0.881 [0.869, 0.893]	0.951 [0.943, 0.960]	0.897 [0.884, 0.910]	0.869 [0.851, 0.888]	0.883 [0.871, 0.894]
Support Vector Machine	0.870 [0.862, 0.878]	0.944 [0.938, 0.950]	0.853 [0.845, 0.861]	0.884 [0.868, 0.899]	0.868 [0.859, 0.876]
Gradient Boost Machine	0.896 [0.884, 0.908]	0.962 [0.957, 0.967]	0.886 [0.870, 0.901]	0.904 [0.890, 0.917]	0.895 [0.882, 0.907]
Multi-Layer Perceptron	0.914 [0.893, 0.935]	0.945 [0.939, 0.950]	0.911 [0.891, 0.931]	0.917 [0.894, 0.940]	0.914 [0.893, 0.935]
Long Short-Term Memory Neural Network	0.939 [0.905, 0.972]	0.959 [0.949, 0.969]	0.943 [0.909, 0.977]	0.935 [0.903, 0.967]	0.939 [0.906, 0.972]

Supplementary Table 2. Summary of cross-validation results for model performance on the validation set in hypertension mortality prediction using top 30 features.

**Models / Metrics**	**Accuracy, 95% CI**	**AUROC, 95% CI**	**Sensitivity, 95% CI**	**Specificity, 95% CI**	**F1 Score, 95% CI**
Logistic Regression	0.861 [0.849, 0.873]	0.938 [0.932, 0.945]	0.840 [0.828, 0.852]	0.876 [0.859, 0.894]	0.858 [0.843, 0.872]
Random Forest	0.875 [0.863, 0.887]	0.946 [0.940, 0.952]	0.882 [0.867, 0.897]	0.869 [0.854, 0.884]	0.876 [0.863, 0.889]
Support Vector Machine	0.866 [0.851, 0.881]	0.940 [0.934, 0.947]	0.849 [0.838, 0.861]	0.879 [0.857, 0.901]	0.864 [0.847, 0.880]
Gradient Boost Machine	0.892 [0.884, 0.899]	0.960 [0.956, 0.964]	0.886 [0.873, 0.898]	0.897 [0.886, 0.907]	0.891 [0.882, 0.900]
Multi-Layer Perceptron	0.871 [0.863, 0.880]	0.932 [0.927, 0.938]	0.868 [0.864, 0.872]	0.873 [0.858, 0.889]	0.871 [0.862, 0.879]
Long Short-Term Memory Neural Network	0.881 [0.862, 0.899]	0.935 [0.926, 0.944]	0.889 [0.875, 0.903]	0.875 [0.851, 0.898]	0.882 [0.863, 0.900]

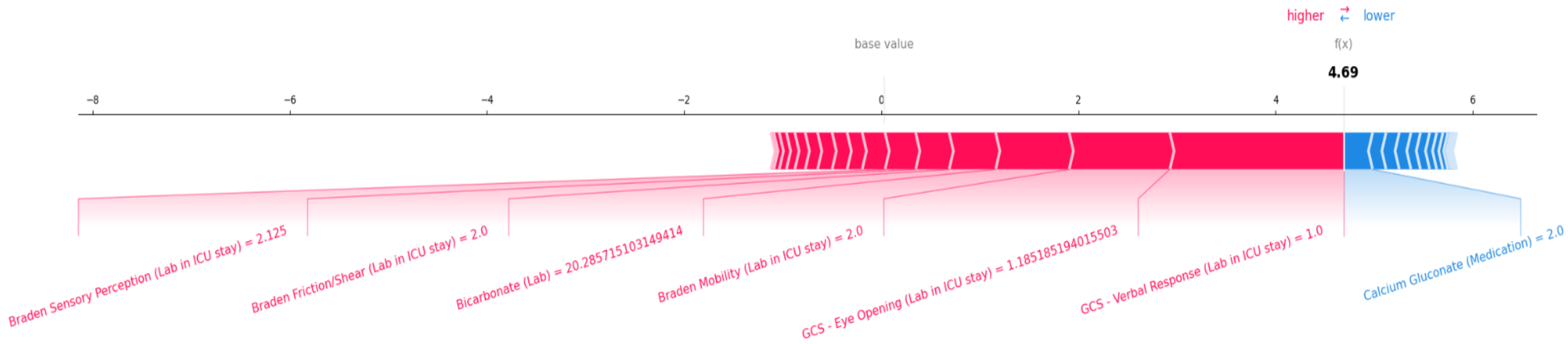

Supplementary Figure 1. Local explanation example for the positive (mortality) case.

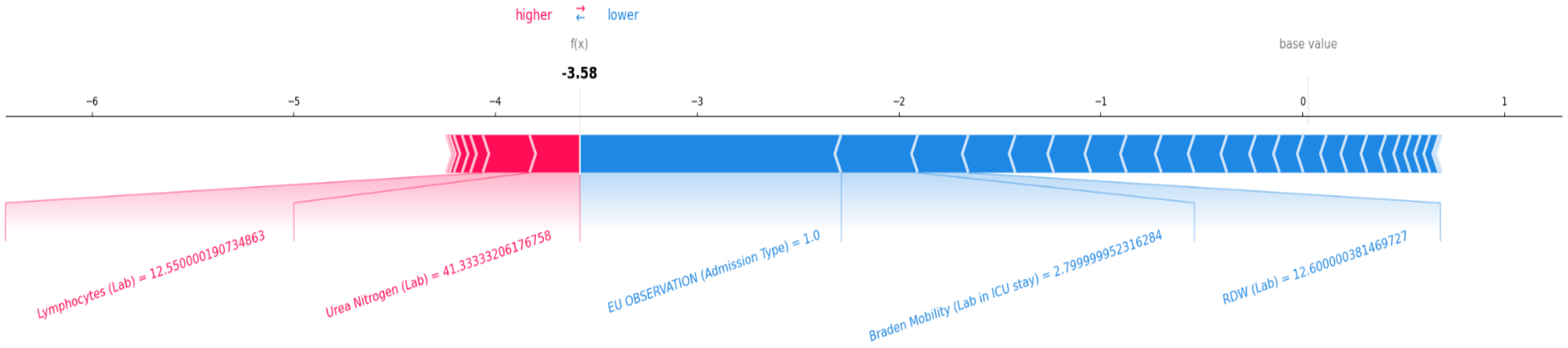

Supplementary Figure 2. Local explanation example for the negative (non-mortality) case.
